# Supplementary material for: Visfatin Facilitates VEGF-D-Induced Lymphangiogenesis through Activating HIF-1α and Suppressing miR-2277-3p in Human Chondrosarcoma
Source: Int J Mol Sci. 2024 May 9;25(10):5142. doi: 10.3390/ijms25105142 (PMC11121249; doi:10.3390/ijms25105142)
Supplement: Supplementary file 1 [file ijms-25-05142-s001.zip › ijms-2970003-supplementary.pdf]

Supplementary data

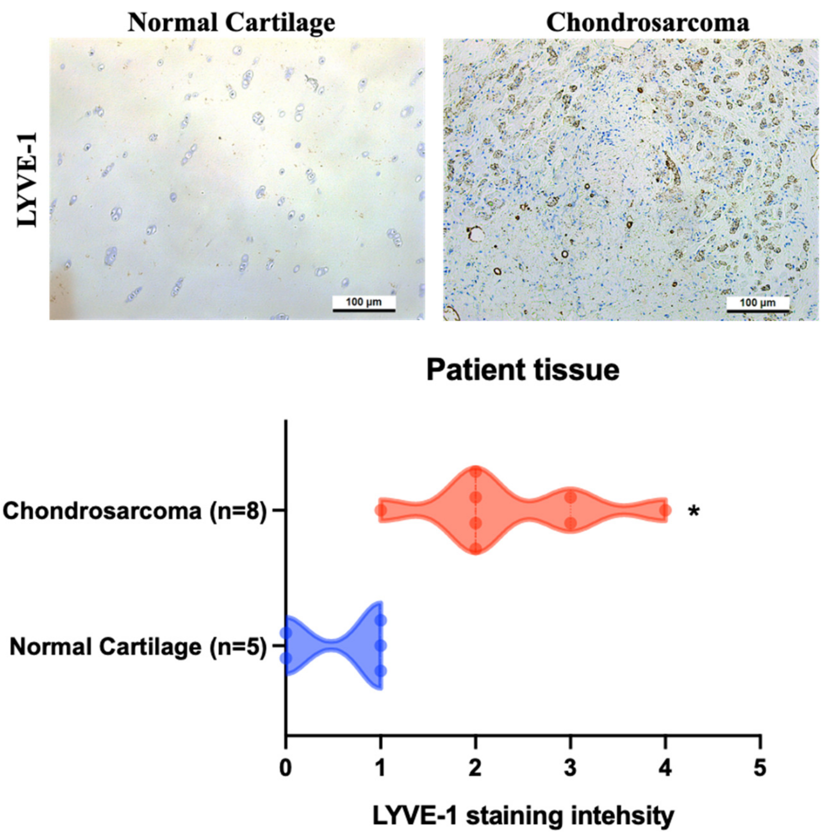

Figure S1. Higher levels of LYVE-1 in chondrosarcoma patients. IHC staining was performed for LYVE-1 levels in chondrosarcoma patients, followed by photography and quantification. * $p < 0.05$ versus normal cartilage.

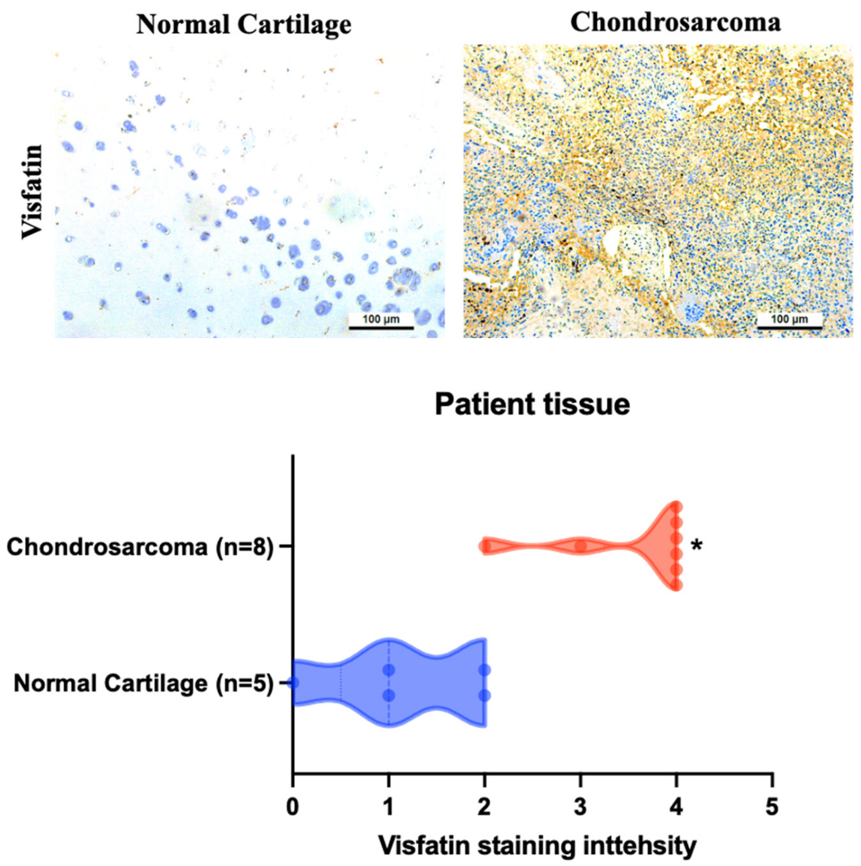

Figure S2. Higher levels of visfatin in chondrosarcoma patients. IHC staining was performed for visfatin levels in chondrosarcoma patients, followed by photography and quantification. * $p < 0.05$ versus normal cartilage.

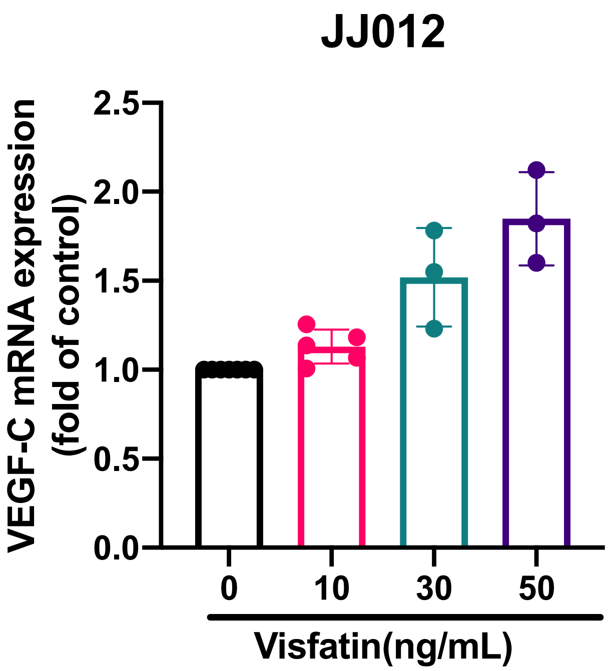

Figure S3. Visfatin increases VEGF-C expression in chondrosarcoma. JJ012 cells were treated with visfatin, the VEGF-C mRNA expression was examined by qPCR.

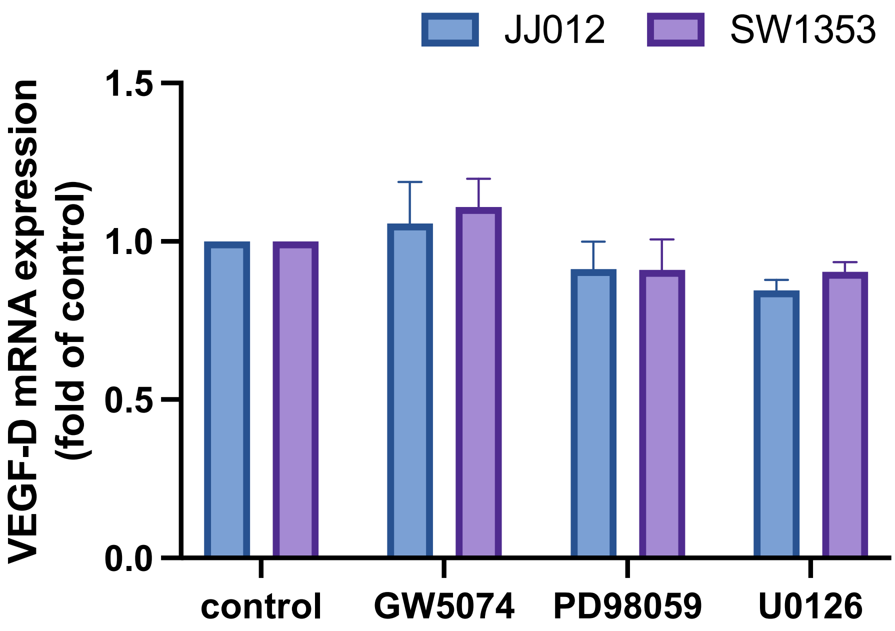

Figure S4. The pharmacological inhibitors did not affect basal levels of VEGF-D. JJ012 cells were treated with indicated inhibitors, the VEGF-D mRNA expression was examined by qPCR.

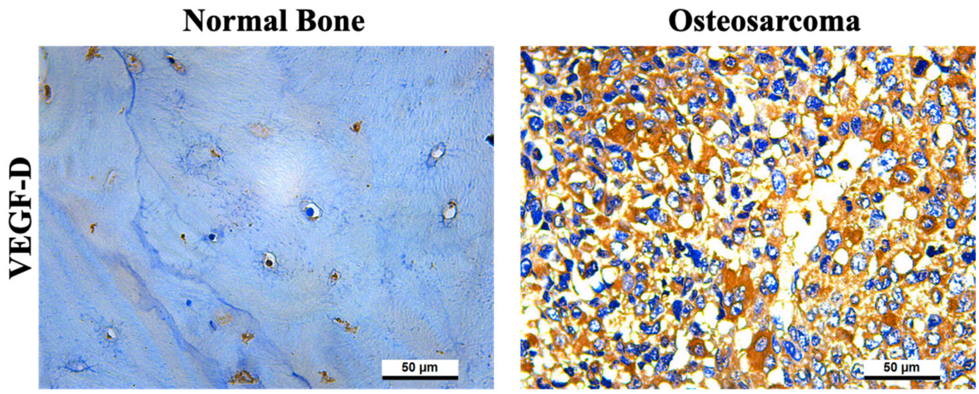

Figure S5. Higher levels of VEGF-D in osteosarcoma patients. IHC staining was performed for VEGF-D levels in osteosarcoma patients, followed by photography.
